# Antigen-Presenting Cells in Psoriasis

**DOI:** 10.3390/life12020234

**Published:** 2022-02-03

**Authors:** Dóra Antal, Shahrzad Alimohammadi, Péter Bai, Attila Gábor Szöllősi, Magdolna Szántó

**Affiliations:** 1 Department of Medical Chemistry, Faculty of Medicine, University of Debrecen, 4032 Debrecen, Hungary; antal.dora@med.unideb.hu (D.A.); baip@med.unideb.hu (P.B.); 2 Department of Immunology, Faculty of Medicine, University of Debrecen, 4032 Debrecen, Hungary; shahrzad.alimohammadi@med.unideb.hu (S.A.); szollosi.attila@med.unideb.hu (A.G.S.)

**Keywords:** psoriasis, antigen-presenting cells, autoantigens, keratinocyte, mast cell, neuropeptide

## Abstract

Psoriasis is classically considered a chronic inflammatory skin disorder, however the identification of autoantigens in its pathogenesis established it as a T cell mediated autoimmune disease. As such professional antigen-presenting cells (APCs) are key players in the development of lesions. APCs in the skin include dendritic cells, Langerhans cells and monocytes/macrophages. In addition, epidermal keratinocytes and dermal mast cells are also endowed with antigen-presenting capacity. Skin APCs have central role in the maintenance of cutaneous immune homeostasis, as well as in initiating and sustaining inflammation under pathologic conditions. In this review we discuss the functional specialization of human skin APCs that promote T cell activation and adaptive immune response during psoriasis initiation and onset.

## 1. Introduction

Psoriasis is a chronic, relapsing-remitting skin disorder that affects about 2% of the world’s population. The clinical phenotype of the most common plaque-type psoriasis presents well-demarcated, scaly, inflamed patches, typically on knees, elbows, or scalp [1]. The etiology of psoriasis is complex, with environmental and genetic factors contributing to disease development. Psoriasis is considered a mixed auto-inflammatory and autoimmune skin disease with the involvement of both innate and adaptive arms of the immune system in its pathogenesis [2]. In addition, the nervous system contributes to disease development and maintenance through the release of neuropeptides that may modify the function of immune cells in the skin [3].

There are multiple genetic risk variants that has been associated with psoriasis. Among them, the first identified was HLA-C*06:02 [4], which still remains the most strongly associated psoriasis susceptibility allele [5,6]. HLA-C*06:02 encoded protein belongs to the major histocompatibility complex (MHC) class I molecules and plays an important role in presenting cytoplasmic antigens to CD4^+^ and CD8^+^ T cells [7]. In addition, other genes involved in antigen presentation were identified as psoriasis genetic risk factors, such as the ERAP1 and ERAP2, which encode amino-peptidase enzymes that can cleave peptide antigens before presentation to T cells [8]. T cells, and specifically CD8^+^ T cells have been recognized as the critical players in psoriasis pathogenesis [9,10]. CD8^+^ T cells are abundant in psoriasis lesions forming multiple clones [11], suggesting that these cells respond to a set of locally presented antigens. To date, four psoriasis-associated autoantigens have been described: self-nucleic acid complexes of the cathelicidin antimicrobial peptide (LL37 or CAMP) [12], the melanocytic antigen ADAMTS-like protein 5 (ADAMTSL5) [13], the lipid antigen PLA2G4D [14] and keratin 17 [15], which may trigger autoimmune responses in psoriasis. LL37, ADAMTSL5 and keratin 17 may be presented by HLA-C molecules, while CD1a^+^ Langerhans cells may present PLA2G4D to the T cell receptors (TCR) of reactive CD4^+^ or CD8^+^ T cells [16,17]. As a consequence, activated T cells release pro-inflammatory cytokines, including interleukin (IL)17A, tumor necrosis factor (TNF)α, interferon (IFN)γ and IL22 [9,18] that act on keratinocytes, which sustain and propagate the chronic inflammatory process by producing pro-inflammatory cytokines, chemokines, and antimicrobial peptides, driving the psoriatic inflammatory circuit. The above findings well illustrate that antigen presentation plays a key role in psoriasis pathomechanism. Professional antigen-presenting cells (APCs) in the skin involve different dendritic cell (DC) subsets, Langerhans cells and macrophages [19]. In addition, epidermal keratinocytes and mast cells are also capable to antigen presentation by expressing MHC class I [20], and under certain conditions even MHC class II [21,22,23] molecules. This concise review will consider only human data regarding the functional role of APCs in psoriasis.

## 2. Professional and “Non-Professional” Antigen-Presenting Cells in the Psoriasis Pathomechanism

### 2.1. Professional APCs

#### 2.1.1. Plasmacytoid Dendritic Cells

Plasmacytoid dendritic cells (pDCs) represent a specialized, type I IFN-producing DC subset that have a major role in antiviral response [24]. pDCs display a unique toll-like receptor (TLR) profile by expressing TLR7 and TLR9 [25], by which pDCs recognize viral and microbial nucleic acid motifs [26]. Under normal conditions, pDCs circulate in peripheral blood and are present in lymphoid tissues [27,28], but not in healthy skin [29]. However, in case of infection, injury or autoimmunity, pDCs can infiltrate the dermis and the basal layer of the epidermis [29,30]. pDCs were detected both in lesional and uninvolved skin of psoriatic patients, and pDC-derived IFNα was shown to be essential for the early developmental stage of psoriasis by promoting the maturation of myeloid DCs, and inducing T cell expansion in pre-psoriatic skin [30]. In contrast to myeloid DCs, blood pDCs of psoriasis patients are dormant, and function only in the psoriatic skin milieu [31]. Interestingly, pDCs are absent in atopic dermatitis lesions, suggesting a unique role for pDCs in psoriasis pathogenesis [29,30,32]. In classical psoriasis, pDC-derived IFNα activates myeloid DCs to produce TNF and IL23. TNF, in turn, induces maturation of pDCs, which limit their IFNα secretion [33]. In the absence of TNF, maturation of pDCs decreases, and their IFNα production is extended, driving a phenomenon called paradoxical psoriasis [33,34], which was introduced as a side effect of anti-TNF therapy. Psoriasis may also be exacerbated in patients under treatment with imiquimod that is a strong inducer of TLR7 in pDCs [32]. The molecular players of pDC activation in psoriasis was getting characterized over the past decade. Dermal expression of the chemotactic factor chemerin may be an early event leading to pDC recruitment to the developing psoriatic skin [35]. During later stages, in chronic plaques, chemerin expression is markedly lower, which coincides with the reduced number of pDCs [35]. Under normal conditions, host-derived self-nucleic acids released by apoptotic or necrotic cells cannot activate pDCs. However, in the presence of antimicrobial peptides (AMP), such as LL37 (or CAMP), B-defensins and Ribonuclease 7, inert self-DNA can be converted into potent autoantigen triggering IFNα production by pDCs, and driving autoimmunity in psoriasis [12,36,37,38]. Of note, AMPs are overexpressed in psoriasis and, consequently, pDC activation is sustained [37]. Post-translational modifications also seem to influence IFNα production by pDCs. The palmitoylating agent Zdhhc2 is required for pDC accumulation in psoriatic skin by controlling the phosphorylation of IRF7 that is a key transcription factor regulating IFNα production of pDCs [39]. The H4 histamine receptor is highly expressed on pDCs in psoriasis and histamine regulates the cytokine production and migration of pDCs [40], supporting the role of neuroimmune interactions in psoriasis progression.

#### 2.1.2. Dermal Myeloid Dendritic Cells

The majority of resident DCs in the skin are of myeloid origin and are located in the dermis [41]. In steady-state healthy skin, the majority of dermal myeloid DCs express CD1c, also known as blood DC antigen (BDCA) 1, and CD11c, which are useful markers to distinguish dermal DCs from macrophages [42]. In psoriasis, however, CD11c^+^ CD1c^−^ “inflammatory” dermal DCs dominate [43]. In the psoriatic skin, dermal DCs are activated by IFNα released by pDCs, by LL37-self-DNA/RNA autoantigens through TLR8 expression, as well as by keratinocyte-derived pro-inflammatory cytokines such as TNF, IL6, and IL1β [44,45]. Inflammatory DCs may produce TNF, inducible nitric oxide synthase (iNOS), IL12, IL20, and they are the major sources of IL23 in psoriatic lesions [43]. IL20 promotes proliferation of keratinocytes [46], IL23 induces terminal differentiation and IL17 production of CD4^+^ T helper (Th)17 and CD8^+^ T cells [47], and IL12 may polarize CD4^+^ Th1 cells [42]. Pro-inflammatory Th17 and Th1 cytokines then act on keratinocytes, propagating chronic inflammation. In addition, LL37 and ADAMTSL5 antigens are coexpressed with DCs in lesional skin, suggesting that DCs are involved in the presentation of these antigens to auto-reactive T cell populations [48]. 

#### 2.1.3. Langerhans Cells

Langerhans cells, first described by Paul Langerhans in 1868 and mistakenly identified as members of the peripheral nervous system, have since been accepted as dendritic cells located in the epidermis [49], which makes them the only professional APC in the steady-state in the human epidermis. Interestingly, although originally considered a typical example of migratory dendritic cells, the exact classification of these cells as resident macrophages (based on their ontogeny) or dendritic cells (based on their function) is still not settled [50]. 

Their role in psoriasis is also debated, as certain publications have reported decreased [51,52,53], increased [54,55,56], and even unchanged numbers of LCs in psoriatic lesions [57,58,59,60]. The differences in results stem from a variety of factors, and might be due to the fact that psoriasis is not a completely homogenous disease. By differentiating between early and late-onset psoriasis we are already faced with a marked difference in the role of Langerhans cells in the disease: in early onset psoriasis (generally regarded as starting before 40) the mobilization and migration of LCs from the epidermis severely impaired [61,62], while in late-onset psoriasis keratinocytes do not produce cytokines that inhibit the migration of LCs [63]. 

Pro-inflammatory roles of LCs in psoriasis pathogenesis were found at the molecular level, where there is a possibility for a positive feedback loop to develop between keratinocytes, LCs and T cells. LCs produce IL23 after STAT3 activation in keratinocytes via the p38α signalling pathway [60,64,65]. IL23 produced by LCs stimulates IL17 release from T cells, which leads to changes in keratinocyte secretome resulting in retention of LCs in the epidermis and further IL17 release. The fact that LCs are retained might also promote the development of the disease since migratory LCs can induce tolerogenic responses in draining lymph nodes. Since psoriasis is at least in part an autoimmune disease, this loss of local tolerance could be one of the major contributors to the disease.

Of note, it is not only peptide antigens that can contribute to psoriasis development, but self-lipid antigens as well. One of the primary markers used to distinguish LCs in situ is CD1a, which has been shown to present self-lipid antigens to CD4^+^ T helper (T_H_) cells, especially in psoriatic skin [16]. LCs are under neuropeptide control in the epidermis, as both calcitonin gene-related peptide (CGRP) and vasoactive intestinal peptide (VIP) may suppress antigen presentation by LCs [66].

#### 2.1.4. Macrophages

Macrophages are important effectors of the innate immune system. Primarily, macrophages are phagocytic cells vital to host survival, as macrophages are involved in the clearance of erythrocytes, cellular debris generated during tissue remodelling, as well as apoptotic and necrotic cells [67]. Macrophages specifically express the scavenger receptor CD163 and Factor XIIIA (FXIIIA) in normal skin which distinguish macrophages from resident dermal DCs [68,69]. The immune-stimulatory capacity of CD163^+^ FXIIIA^+^ macrophages is markedly lower than that of dermal DCs [69]. Macrophages are more abundant in psoriatic lesions compared to normal skin as determined by increased expression of CD163 [68], but the exact role of macrophages in psoriasis is not fully understood. LL37 promotes pro-inflammatory macrophage differentiation [70] and CD163^+^ macrophages are co-expressed with LL37 and ADAMTSL5 in both lesional and non-lesional psoriatic skin [48], implying that macrophages, similarly as DCs, take part in the presentation of autoantigens to T cells. Like myeloid DCs, macrophages also have the ability to produce IL23 that may promote activation of CD4^+^ and CD8^+^ T cell populations and contribute to the IL17-mediated inflammatory response is psoriatic skin [71].

A recent article shed some new light on macrophages in psoriasis by revealing a re-emergence of developmental macrophage gene programs in psoriasis [72]. Single-cell RNA-sequencing analyses have been performed from developing healthy fetal skin, adult healthy skin and adult skin with psoriasis. Macrophages of adult skin were classified into Mac1 and Mac2 subtypes based on specific marker signatures [72,73]. Both Mac1 and Mac2 express CD68, while Mac1 is characterized by higher expression of CD163, macrophage receptor with collagenous structure (MARCO), and complement transcripts. Mac2, on the other hand, displays expression of FXIIIA and markers of alternative activation and immune suppression. Adult skin Mac2, which aligned with fetal skin macrophages, was significantly elevated in psoriatic skin, suggesting a role for Mac2 in psoriasis pathogenesis and also a shared cellular program with macrophages of fetal development [72].

### 2.2. Non-Professional APCs in Psoriasis

#### 2.2.1. Keratinocytes

Though the pathogenic role of keratinocytes in psoriasis was long debated, growing evidence suggests that hyperproliferation and aberrant differentiation of keratinocytes is a consequence rather than the cause of immune activation in psoriasis, and keratinocytes play a central role in linking innate and adaptive immune responses during disease manifestation [74]. Keratinocytes facilitate the initiation phase of psoriasis by expressing the chemotactic factor chemerin that promotes pDC migration to pre-psoriatic skin [35]. Certain triggering factors, such as epidermal injury may promote keratinocytes to excessively produce AMPs that have the ability to form complex with otherwise inert self-DNA and thereby act as strong inducers of pDC activation and secretion of IFNα in psoriatic skin [38]. In addition, the cathelicidin AMP LL37 can induce components of innate immunity in keratinocytes by a paracrine or autocrine manner. Among them, the IL1 family member IL36γ is highly expressed in psoriatic skin and increases the production of CXCL8, CXCL1, CXCL10 and CCL20 chemokines, as well as cytokines IL6, G-CSF, and GM-CSF in keratinocytes [75]. These chemoattractants can recruit and activate a burst of immune cells, such as mDCs, macrophages, neutrophils, T cells, and Natural Killer cells in the early stage of psoriasis [75]. IL36γ may also lead to proliferative responses in keratinocytes [44]. Furthermore, keratinocytes are the main cell type in psoriasis that express IL17R, the receptor for IL17 [74], and the keratinocyte IL17 gene set is enriched in the psoriasis transcriptome [76]. IL17 induce proliferation and further AMP (e.g., defensins, cathelicidins) and pro-inflammatory cytokine (e.g., IL6, IL8, TNF) production in keratinocytes, and these products act back on DCs and T cells, creating a self-amplifying loop [77]. Moreover, keratinocyte-produced IL23 is, in itself, sufficient to induce IL17 production of T cells and cause chronic skin inflammation [78]. Keratinocytes are important sources of angiogenic factors and the autoantigen keratin 17, as well [79]. Keratinocytes may be involved in the presentation of soluble antigens to CD8^+^ T cells through HLA-C molecules (Liang, 2017), which may be a central driver of inflammatory circuits in plaque psoriasis. Keratinocytes are targeted by neuropeptides in psoriatic lesions. Serotonin stimulates keratinocyte proliferation and differentiation through the 5-HT3 that is located in the basal layer of the epidermis in lesional skin of psoriasis [80]. SP can also induce proliferation and pro-inflammatory cytokine (e.g., IL1, IL8, TNFα) synthesis in keratinocytes [81]. 

#### 2.2.2. Mast Cells

Mast cells (MCs) are tissue-resident effectors of innate immune responses that have a well-defined role in allergic responses. In the skin, MCs are found in the upper dermis, in close vicinity to DCs [82]. A role for MCs in psoriasis has long been postulated [83], but consecutive studies are still missing. MCs can be activated by neuropeptides [84]. SP induces vascular endothelial growth factor (VEGF) release from MCs that is increased in psoriasis [85]. MCs were found as important contributors to IL17 production in psoriatic lesions [86]. IFNγ–primed human mast cells can induce the generation of IL22-producing T_H_ cells from CD4^+^ memory T cells, and this process was demonstrated in psoriasis [87]. A recent immune cell infiltrate analysis of psoriatic lesional skin revealed that receptor tyrosine kinase (c-Kit) and tryptase double-positive, activated MCs are enriched in psoriatic skin, and their number gradually decreases upon clinical treatment, supporting an important role of MC activation in the progression of psoriasis [88].

MCs express MHC class II [23,89] and can act as non-professional APCs [90]. Baseline or IFNγ-primed MCs can take up both soluble and particulate antigens and present them to autologous CD4^+^ T cells [90]. Whether antigen presentation by MCs is significant in the pathomechanism of psoriasis remains to be elucidated. It is of note though, that LL37, which may act as an autoantigen in psoriasis, can directly induce pro-inflammatory cytokine synthesis in MCs [91]. 

## 3. Summary and Future Perspectives

**Figure 1:**
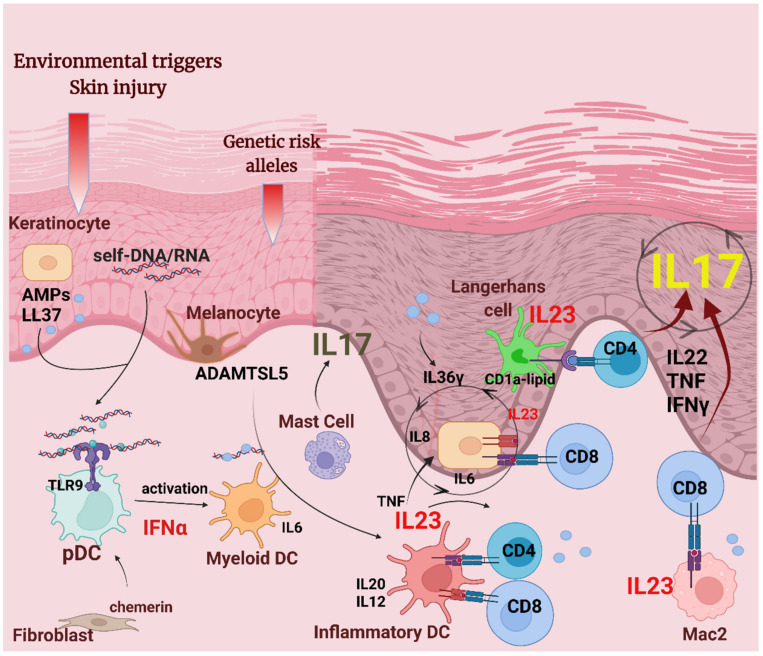
APCs in psoriasis pathogenesis. Environmental factors, such as epidermal injury, together with psoriasis susceptibility genes, are involved in the pathogenic mechanisms leading to psoriasis initiation. In the initiation phase, damaged keratinocytes release self-nucleic acids and AMPs, such as LL37. LL37/self-nucleic acid complexes and the chemotactic factor chemerin recruit plasmacytoid dendritic cells (pDCs) that release IFNα, leading to the activation of dermal myeloid DCs and inflammatory dermal DCs that produce cytokines IL20, IL12, and IL23. Autoantigens LL37 and ADAMTSL5 may be presented on HLA-C (MHC class I) by DCs, keratinocytes, and macrophages to CD4^+^ and CD8^+^ T cells. Activated T cells will produce pro-inflammatory cytokines IL22, TNF, IFNγ, and IL17 that act on keratinocytes to induce the proliferation and production of AMPs and keratinocyte-derived pro-inflammatory cytokines. IL23 secreted by keratinocytes give feedback on T cells and drive a sustained, chronic skin inflammation. Activated mast cells have a share in IL17 production in psoriatic plaques. Macrophages and Langerhans cells also contribute to the psoriatic milieu by presenting cytosolic antigens to reactive T cells, and producing IL23.

Our perception of APCs in pathological skin conditions has greatly improved in the past decade, owing, in part, to a more accurate definition of surface markers of different APC subsets under steady-state and inflammatory milieu. In parallel, appreciation of the central role of APCs and autoimmune processes in the pathomechanism of psoriasis is constantly growing (Figure 1). Naturally, development of psoriasis cannot simply be explained by autoantigen presentation in predisposed individuals. The fine-tuning of the complex interplay between the innate and adaptive arms of the immune system determines the actual clinical manifestation of psoriasis. Data supports that innate mechanisms are more dominant in patients with severe outcome and systemic comorbidities, while in mild disease mechanisms of adaptive immunity are more important [92]. A better understanding of the functional programming of the APC network in the psoriatic microenvironment in different disease stages and manifestations may be vital in the development of novel therapeutic targets. This is of importance not only in the case of psoriasis, but in other inflammatory and autoimmune diseases that share similar pathogenetic pathways.
